# Influence of TiO_2_ and ZrO_2_ Nanoparticles on Adhesive Bond Strength and Viscosity of Dentin Polymer: A Physical and Chemical Evaluation

**DOI:** 10.3390/polym13213794

**Published:** 2021-11-02

**Authors:** Samar Al-Saleh, Abdullah Alateeq, Abdulaziz H. Alshaya, Amal S. Al-Qahtani, Huda I. Tulbah, Mashael Binhasan, Sara Shabib, Imran Farooq, Fahim Vohra, Tariq Abduljabbar

**Affiliations:** 1 Department of Prosthetic Dental Sciences, College of Dentistry, King Saud University, P.O. Box 21069, Riyadh 11475, Saudi Arabia; salsaleh@ksu.edu.sa (S.A.-S.); aalkahtany@ksu.edu.sa (A.S.A.-Q.); htulba@ksu.edu.sa (H.I.T.); fvohra@ksu.edu.sa (F.V.); 2 College of Dentistry, King Saud University, P.O. Box 21069, Riyadh 11475, Saudi Arabia; alateeq.ay@gmail.com (A.A.); shaya.abdulaziz@gmail.com (A.H.A.); 3 Department of Restorative Dentistry, Division of Operative Dentistry, College of Dentistry, King Saud University, Riyadh 60169, Saudi Arabia; mbinhasan@ksu.edu.sa (M.B.); sashabib@ksu.edu.sa (S.S.); 4 Faculty of Dentistry, University of Toronto, Toronto, ON M5G 1G6, Canada; imran.farooq@mail.utoronto.ca

**Keywords:** adhesive, dentin, bonding, zirconium, titanium, microscopy

## Abstract

The present study aimed to formulate an experimental adhesive (EA) and reinforce it with 5 wt.% titanium dioxide (TiO_2_) or zirconium oxide (ZrO_2_) to yield 5% TiO_2_ and 5% ZrO_2_ adhesives, respectively, and then analyze the impact of this reinforcement on various mechanical properties of the adhesives. The EA contained a blend of monomers such as bisphenol A glycol dimethacrylate (BisGMA), triethylene glycol dimethacrylate (TEGDMA), 2-hydroxyethyl methacrylate (HEMA), and ethyl 4-dimethylamino benzoate and camphorquinone. The EA included ethyl 4-dimethylamino benzoate and camphorquinone photo-initiators, and diphenyliodonium hexafluorophosphate (DPIHP) was also included to act as an electron initiator. The TiO_2_ and ZrO_2_ nanoparticles were incorporated into the EA post-synthesis. To characterize the filler nanoparticles, scanning electron microscopy (SEM) and line-energy dispersive X-ray (EDX) spectroscopy were performed. The adhesives were characterized by analyzing their rheological properties, shear-bond strength (SBS), and interfacial failure types. Further, the resin–dentin interface was also analyzed via SEM. The TiO_2_ nanoparticles were spherically shaped on the SEM micrographs, while the ZrO_2_ nanoparticles were seen as non-uniformly shaped agglomerates. The EDX mapping demonstrated the presence of Ti and oxygen for TiO_2_ and Zr and oxygen for the ZrO_2_ nanoparticles. Both 5% TiO_2_ and 5% ZrO_2_ adhesives revealed decreased viscosity as compared with the EA. The 5% TiO_2_ adhesive demonstrated higher SBS values for both non-thermocycled (NTC) and thermocycled samples (NTC: 25.35 ± 1.53, TC: 23.89 ± 1.95 MPa), followed by the 5% ZrO_2_ adhesive group (NTC: 23.10 ± 2.22, TC: 20.72 ± 1.32 MPa). The bulk of the failures (>70%) were of adhesive type in all groups. The SEM analysis of the resin–dentin interface revealed the development of a hybrid layer and resin tags (of variable depth) for the EA and 5% TiO_2_ groups. However, for the 5% ZrO_2_ group, the hybrid layer and resin tag establishment appeared compromised. Reinforcement of the EA with TiO_2_ or ZrO_2_ caused an increase in the adhesive’s SBS (with the 5% TiO_2_ group demonstrating the highest values) in comparison with the EA (without nanoparticles). However, both nanoparticle-containing adhesives revealed decreased viscosity compared with the EA (without nanoparticles). Further studies investigating the impact of diverse filler concentrations on the properties of adhesives are suggested.

## 1. Introduction

Adhesion is described in the literature as the sticking or attachment of two surfaces to each other [1]. The concept of adhesion has revolutionized dentistry [2] as it has led to the development of restorative materials that involve minimal intervention [3]. Dentin adhesives are one such material that were introduced in the late 1940s, but since then, they have been modified in order to optimize their function [4]. Regrettably, the bond formed between the adhesive and dentin tissue is unstable and can lose strength with prolonged use, resulting in the restoration’s failure [5]. There are several reasons for this gradual decline in the bond strength of dentin adhesive. One reason pertains to the composition of dental tissue: adhesion to dentin is more complex, when compared with enamel, as dentin is collagenous, hydrophilic, and ever-regenerating [1]. Another reason is the conventional composition of dentin adhesives. This composition plays a role in failure due to breaking of the covalent polymeric bonds of the material via hydrolytic degradation, causing a loss of bond strength and the eventual failure of the adhesive bond [6]. To address this, researchers have made attempts to improve dentin adhesives by adding bioactive inorganic fillers that could improve the bond strength of the adhesives [7]. Previous studies revealed that the addition of inorganic fillers such as hydroxyapatite (HA), silica (SiO_2_), and graphene oxide (GO) can augment the mechanical properties of dentin adhesives [8,9,10]. However, the quest for a filler material that can boost the mechanical properties of dentin adhesive—more than alternative materials—continues.

Titanium dioxide (TiO_2_) is a trace element that is primarily added to different foods because of its high refractive index (RI) [11]. TiO_2_ is also a thermally stable material and is able to resist chemical attacks [12]. Furthermore, TiO_2_ nanoparticles have a large surface area (due to their nanosize) and possess high biocompatibility [13]. In dentistry, TiO_2_ nanoparticles have been used to improve the fracture resistance of endodontically treated teeth [14], osseointegration of dental implants [15], antibacterial potential of a material [13], and bonding to human teeth by incorporating them in the adhesives [16]. In a recent study, the addition of TiO_2_ nanoparticles in experimental dental adhesives demonstrated comparable water sorption, solubility, and biocompatibility with unaltered adhesives, advocating the use of these particles in adhesive dentistry [17]. In another study, it was reported that self-adhesive resin cements containing TiO_2_ displayed a superior degree of conversion (DC) to their unaltered counterparts [18]. The above-mentioned studies verify the potential of TiO_2_ nanoparticles as fillers in dentin adhesives. A major concern with the use of TiO_2_ is their potential cytotoxicity, which is associated with the human body being exposed to it in large quantities [19]. The quantity of TiO_2_ included in dentin adhesives generally ranges from 5 to 10 wt.%, which makes the adhesives less likely to cause any cytotoxicity. Therefore, the potential advantages of the inclusion of TiO_2_ still outweigh its problems.

Zirconium oxide (ZrO_2_) was introduced as a hip replacement prosthesis material and as an alternative to alumina or titanium [20]. ZrO_2_ possesses high biocompatibility and admirable aesthetic and mechanical properties [20]. In dentistry, Zr-based materials are commonly used in the manufacturing of dental prostheses [21], dental implants [22], and yttria-stabilized tetragonal zirconia polycrystal (Y-TZP) endodontic posts [23]. In a previous study, zirconia-based fillers resulted in the improvement of tensile bond strength and toughness of composites [24]. In another study, it was confirmed that the incorporation of Zr-based nanoparticles at 5, 10, 15, and 20 wt.% in the dental adhesive resulted in an increased bond strength with dentin (with higher nanofiller concentrations causing a more efficient increase in the bond strength) [25]. The potential of Zr-based nanoparticles to be used as filler in dentin adhesives is evident from the aforementioned studies, and it would thus be of interest to investigate the influence of their addition on various properties of dentin adhesives. A concerning property of Zr-based materials is the fact that ZrO_2_ degrades at lower temperatures (aging) [26]. However, one factor that influences its degradation is the grain size (larger-sized particles cause degradation and smaller sized particles improve its stability) [27]. As only nanosized ZrO_2_ particles are used in dentin adhesives, the potential to achieve improved bond strength in ZrO_2_-containing adhesives and their beneficial properties advocate their use in dentin adhesives.

Therefore, the aim of the current study was to prepare an experimental adhesive and analyze the impact of reinforcing it with two different nanoparticle groups (TiO_2_ and ZrO_2_). The influence of the integration of TiO_2_ and ZrO_2_ nanoparticles in the EA was studied by analyzing the rheological properties and shear-bond strength (SBS) of the adhesives. We hypothesized that the insertion of these filler nanoparticles would improve rheological properties, SBS, and dentin interaction of the adhesives.

## 2. Materials and Methods

The present study received approval from the institutional review board and ethics committee at King Saud University with approval no. E-20-5545. Furthermore, it was designed according to the principles stated in the Helsinki Declaration of 1964 and its later amendments, and we carefully applied all of the associated ethical protocols. The maxillary premolar teeth used in the experiments of this study were collected from the Oral Surgery Clinics of the Dental Hospital of King Saud University, Riyadh, Saudi Arabia, after obtaining written informed consent from the patients. Only the teeth which were restoration or defect-free were included in this study. These teeth were stored in 10% formalin and used within 30 days post-collection for the experiments.

### 2.1. Preparation of the EA and Its Reinforcement with Filler Nanoparticles

We prepared the EA by following the previous recommendations of Almutairi et al. [10]. Both of the filler nanoparticles were commercially acquired (TiO_2_: particle mixture-634662-Merck SA, Darmstadt, Germany; and ZrO_2_: 643028-Merck SA, Darmstadt, Germany). Post-EA synthesis, 5 wt.% of TiO_2_ and ZrO_2_ nanoparticles were added to the EA, and to ensure their homogenized dispersal, they were sonicated in a centrifuge. These newly prepared adhesives (EA, 5% TiO_2_, and 5% ZrO_2_) were stored at 4 °C and used for further experiments within three weeks of their production.

### 2.2. Scanning Electron Microscopy (SEM) and Energy Dispersive X-ray (EDX) Analysis

Characterization of the TiO_2_ and ZrO_2_ filler nanoparticles was carried out utilizing SEM and EDX analysis. A small quantity of these nanoparticles was mounted on the aluminum stubs, coated with gold, and then observed via an SEM (FEI Quanta 250, Scanning Electron Microscope, OR, USA), which operated at an accelerating voltage of 30 kV. These samples were observed at various magnifications (based on convenience). EDX spectroscopy was also implemented to characterize these filler nanoparticles further and assess their elemental distribution.

### 2.3. Assessment of the Rheological Properties of the Adhesives

The adhesives were first characterized by considering changes in their rheological properties using an MCR-72 rheometer (Anton Paar, Graz, Austria). These rheological properties were evaluated using rotation mode in a frequency sweep pre-set state of 8 mm (parallel plate) and 0.25 mm (opening). The samples were assessed over a wide range of angular frequencies, extending between 0.1 and 100 rad/s at 25 °C.

### 2.4. SBS Testing and Interfacial Fracture Types of the Adhesives

Seventy-five teeth (N = 75) were collected and then equally and randomly divided into three groups such that each adhesive group (EA, 5% TiO_2_, and 5% ZrO_2_) received 25 tooth samples (n = 25). Each of these teeth was prepared for bonding, and then SBS testing was carried out following the prior recommendations of Almutairi et al. [10]. Briefly, the dentin surfaces of the tooth samples were first flattened using a slow-speed diamond disc under running water. The exposed dentin surfaces were then polished using a 600-grit silicone paper under running water and the samples were positioned in the machine such that the shearing stamp loaded the compomer cylinder at a 90° angle. The crosshead speed was 1 mm/min and the cell load capability was 1 kN unit failure. A fitted crescent-shaped copper piece guaranteed the safety of the composite cylinder, which was necessary as each specimen provided two experimental interfaces. The SBS testing values were estimated (in megapascals, MPa) by dividing the peak force at the point of failure (in Newtons) by the bonding area in N/mm^2^, as suggested by Kensche et al. [28]. The SBS was evaluated using a universal testing machine (TIRAtest 2720, TIRA GmbH, Schalkau, Germany). Pre-SBS testing, of the twenty-five samples in each group, ten samples were thermocycled (TC), ten remained non-TC (NTC), and the remaining five were tested for the analysis of the resin–dentin interface. For TC specimens, distilled water baths were carried out for 10,000 cycles at 5 °C and 55 °C with a dwell time of 5 s (THE-1100, SD Mechatronik GmbH, Germany). For the NTC specimens, the bonded samples were stored for 1 day in distilled water prior to sectioning.

In the current study, the interfacial bond failure types were also assessed. This examination was conducted with a digital microscope (Hirox KH 7700, Tokyo, Japan). We divided the failure types into three categories: adhesive, cohesive, and/or mixed.

### 2.5. Evaluation of the Resin–Dentin Interface

Five bonded specimens from each adhesive group were first sectioned by means of a slow-speed isomet saw (Buehler Isomet 2000 Precision saw, Lake Bluff, IL, USA). This helped to form 1 x 1 mm beams. SEM and EDX spectroscopy were again employed in this study to analyze the bonded resin–dentin interface. With the help of a polisher (Beuhler Polisher, Lake Bluff, IL, USA), wet polishing of the beams was executed. This step was followed by their washing and placement in an ultrasonic bath (Bandelin Digital-Sigma-Aldrich Darmstadt, Germany) containing distilled water for 5 min. The conditioning of the samples was then performed using 36% phosphoric acid (DeTrey conditioner, Dentsply, PA, USA) followed by their washing with distilled water and sodium hypochlorite (5.25%) and solution immersion (for 15 min). Cleaning of the specimens was then carried out using distilled water, and they were then dehydrated using ethanol solutions of varying concentrations (80–100%). Gold coating of the specimens was achieved, and the samples were then analyzed using an SEM (FEI Quanta 250, Scanning Electron Microscope, OR, USA) to appraise the resin–dentin interface. The SEM was again operated at an accelerating voltage of 30 kV, and a range of magnifications was utilized.

### 2.6. Statistical Analysis

The outcomes of the SBS testing were gathered, computed on excel sheets, and assessed using SPSS-20.0 (IBM, Chicago, IL, USA). These values were used to calculate mean and standard deviations. The normality of the data was first checked via the Kolmogorov–Smirnov test. On observing non-normal distribution, ANOVA and post-hoc multiple comparison non-parametric tests were then applied to further analyze the results. The statistical significance level was set at 1%.

## 3. Results

### 3.1. Outcomes of the Characterization of the Filler Nanoparticles

**Figure 1:**
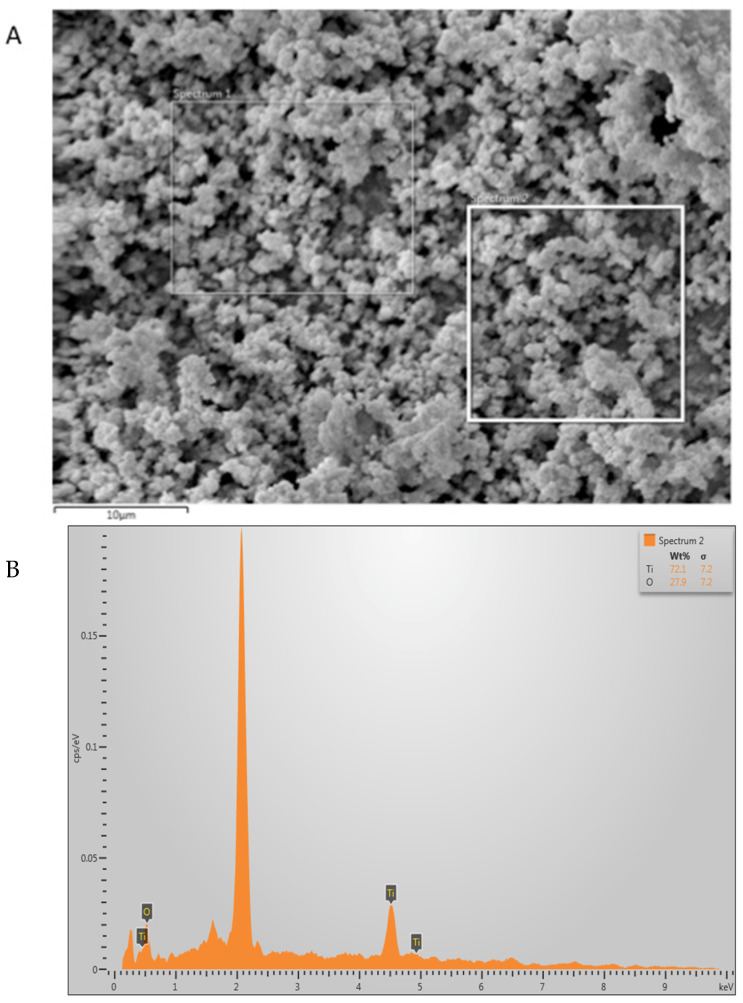
High-magnification (**A**) SEM image of the nano-titanium oxide (TiO_2_) particles. The TiO_2_ powder showed agglomerated particles of various irregularly sized polygonal crystals, ranging from 500 nm to 1000 nm. (**B**) Representative EDX graph showing high percentage of Ti and oxygen.

**Figure 2:**
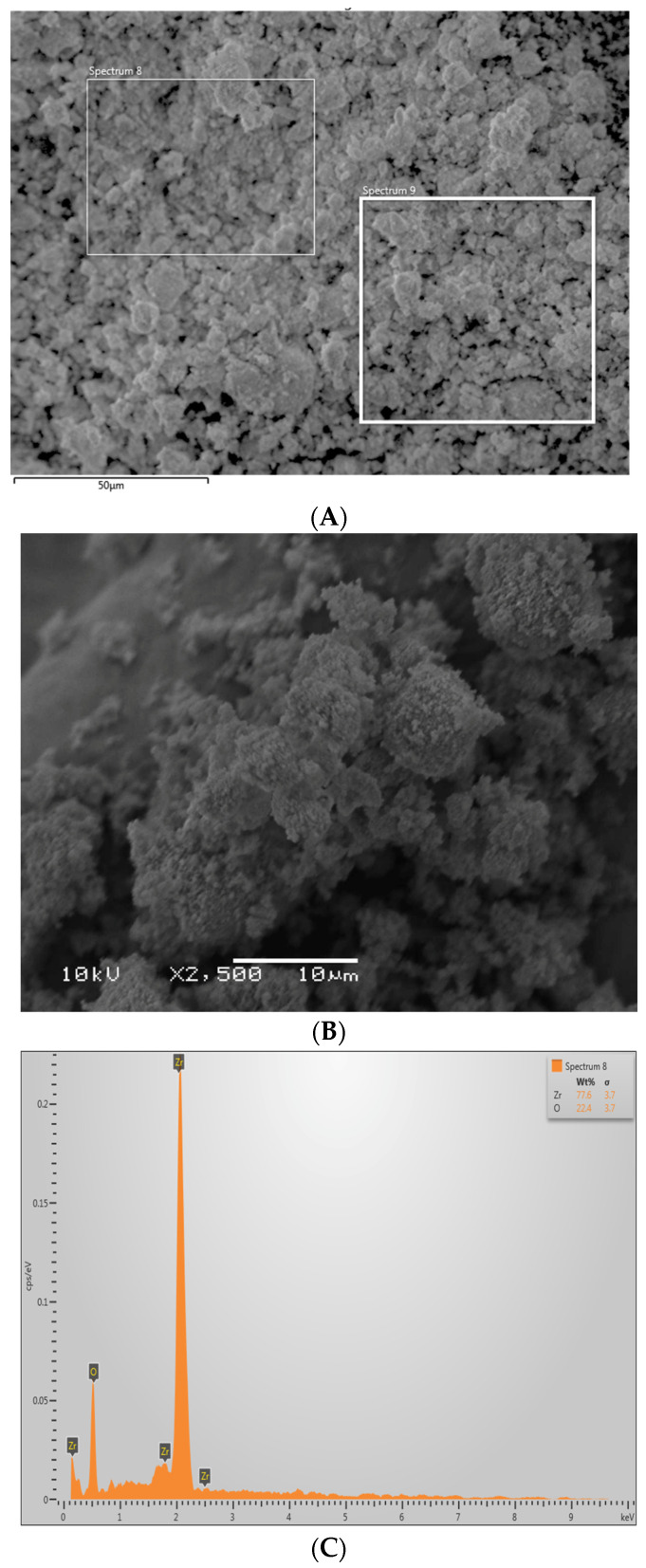
High magnification (**A**,**B**) SEM image of the nano-zirconium oxide (ZrO_2_) particles. The ZrO_2_ powder showed agglomerated particles of irregularly sized crystals around 500 nm. (**C**) Representative EDX graph showing high percentage of Zr and oxygen (O).

The TiO_2_ nanoparticles demonstrated irregular shape on the SEM micrograph (Figure 1A). These filler nanoparticles were spherically shaped in an agglomerated form with an average particle size ranging between 500 and 1000 nm. The EDX mapping verified the occurrence of titanium and oxygen in the TiO_2_ nanoparticles (Figure 1B). The ZrO_2_ nanoparticles were also seen as irregularly shaped on SEM micrographs (Figure 2A,B). These nanoparticles also exhibited agglomerated form, with the particles seen sticking to each other with an average particle size of 500 nm (Figure 2B). The EDX mapping for ZrO_2_ nanoparticles revealed the presence of Zr and oxygen (Figure 2C), which are the two essential elements for ZrO_2_.

### 3.2. Outcomes of the Assessment of the Rheological Properties

**Figure 3:**
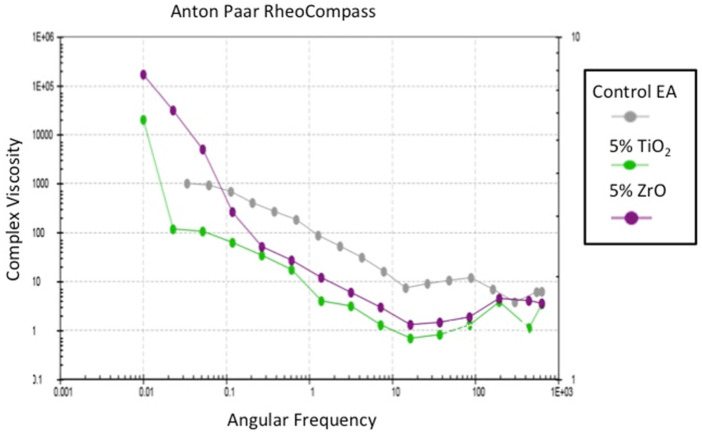
Rheological properties of the experimental control (Control EA), 5%-TiO_2_, and 5% ZrO_2_ adhesives. Complex viscosity is shown for angular frequencies of 0.001 to 1000 rads/s.

The rheological assessment of the adhesives revealed that a decrease in the viscosity was seen when the angular frequency was increased (Figure 3). This decrease was most evident for the 5% ZrO_2_, followed by the 5% TiO_2_. The EA adhesive demonstrated a smaller decrease in the viscosity compared with the other two adhesive groups, although this change in the EA was also noticeable. All three adhesives studied here demonstrated non-Newtonian behavior (shear-thinning or pseudo-plasticity). The observed reduction in viscosity for filler-reinforced adhesive groups could mean that the incorporation of TiO_2_ and ZrO_2_ caused an improved fluidity of the resin/filler mixture; nevertheless, a Newtonian plateau was not witnessed, even at low angular frequencies. In the present study, although the EA group revealed greater viscosity than the other two groups, this trend was not consistently observed, and an overlap was seen between the viscosities of all of the adhesive groups at higher frequencies (Figure 3).

### 3.3. Outcomes of the SBS Testing and Failure Types Investigation

The results of the SBS assessment appeared to be in favor of the 5% TiO_2_ group (both NTC samples: 25.35 ± 1.53 MPa and TC samples: 23.89 ± 1.95 MPa) followed by the 5% ZrO_2_ adhesive group (NTC samples: 23.10 ± 2.22 MPa and TC samples: 20.72 ± 1.32 MPa (Table 1). The EA group revealed the lowest SBS values (NTC samples: 21.03 ± 2.44 MPa and TC samples: 17.62 ± 1.70 MPa) compared with the other two adhesive groups. Statistically significant results (*p* < 0.01) were witnessed upon intergroup comparison when the SBS test values for the NTC samples of the EA group were compared with the other two groups. A similar trend was observed concerning the intergroup comparison of the TC samples, and again the EA group’s SBS values were significantly different (*p* < 0.01) from the other two groups. The intergroup comparison between NTC and TC samples was statistically significant (*p* < 0.01) for all of the adhesive groups (Table 1).

Failures due to adhesion were most commonly found in the present study, accounting for 70 to 100% (in some instances) of the total failures (Table 1). The mixed type was the next most common failure, while cohesive failure was only seen for the NTC samples of the 5% ZrO_2_ group (Table 1).

### 3.4. Outcomes of the Evaluation of the Resin–Dentin Interface

**Figure 4:**
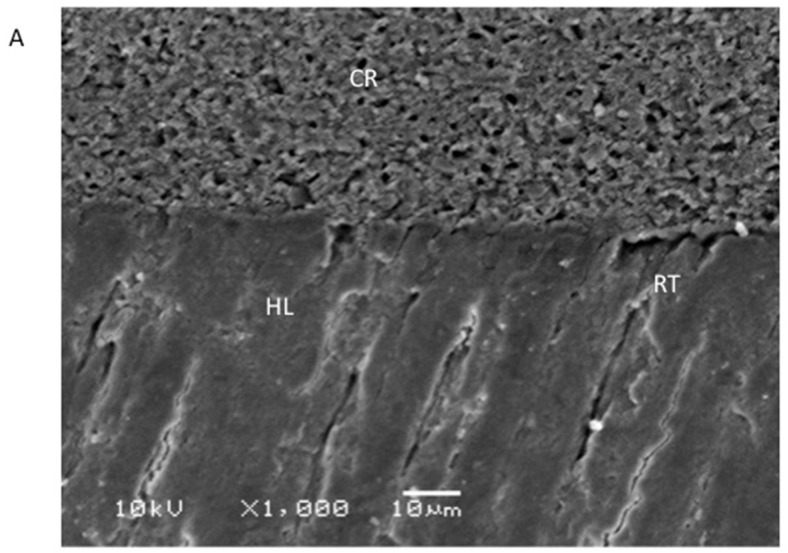

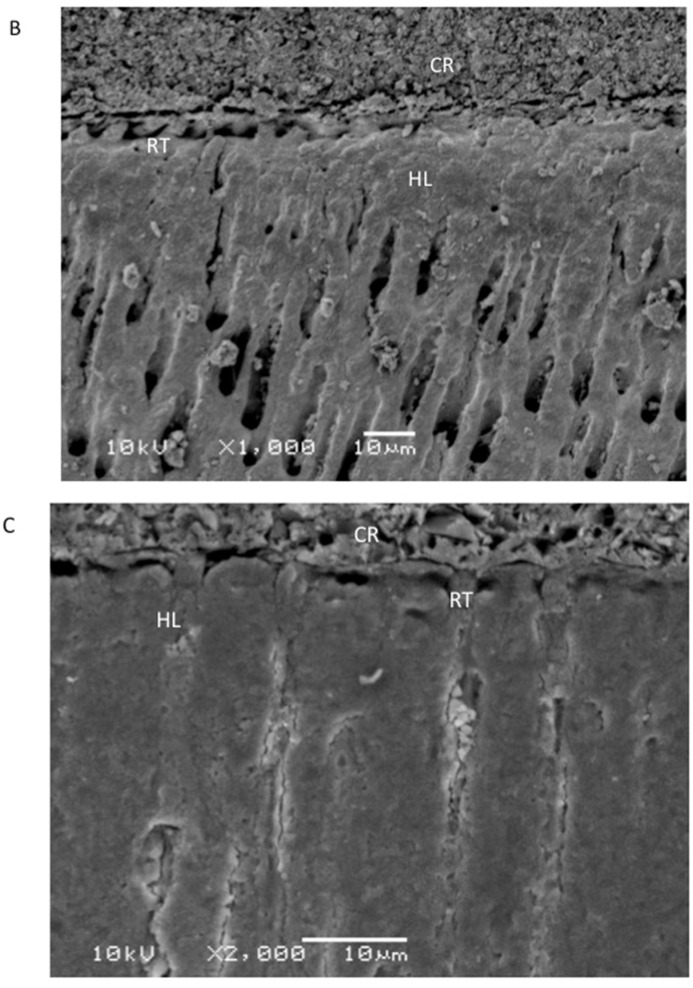
High-magnification images of resin–dentin interface showing: (**A**) composite resin (CR), hybrid layer (HL), and normal resin tag (RT) formation in control samples (EA). (**B**) Normal resin tag formation and standard dentin penetration with TiO incorporated dentin adhesive, (**C**) RT formation and dentin penetration was compromised in ZrO_2_-containing EA (few resin tags).

The SEM analysis revealed the establishment of a hybrid layer and resin tags (of variable depth) for the EA group (Figure 4A). For the 5% TiO_2_ group, again, comparable hybrid layer and resin tag development was seen on the SEM micrograph (Figure 4B). However, for the 5% ZrO_2_ group, it was noted that, although the hybrid layer and resin tag formation was perceived, it was not comparable to the other two groups and seemed to be compromised (Figure 4C).

## 4. Discussion

Based on the results of the current study, we partly accept the hypothesis that the incorporation of filler nanoparticles improves the SBS of adhesives. The hypothesis was also partly rejected, as the 5% TiO_2_ and 5% ZrO_2_ adhesives demonstrated less viscosity as compared with the EA (without filler nanoparticles). Several previous studies in the literature positively affirmed that the integration of inorganic fillers can amplify the mechanical properties of the adhesives studied [8,10,29]. TiO_2_ is mostly used in dentistry to coat the outer surface of dental implants as it offers excellent biocompatibility and remarkable mechanical properties [30]. TiO_2_ is also a strong antibacterial material that reduces the incidence of peri-implantitis (a common problem in dental implantology) [15]. In addition, the inclusion of TiO_2_ as a filler has resulted in an improvement of its physicochemical properties [31], and its inclusion in the composition of adhesive resin cement improved the DC of the material [18]. Similarly, ZrO_2_ is a bioinert and biocompatible material [32]. In dentistry, both Zr and Ti are preferred materials for the fabrication and coating of implants as they do not inhibit the formation and differentiation of osteoblasts, thus promoting osseointegration [33,34]. Zr-based fillers have been shown to result in an improvement to the mechanical properties of dental adhesives [25]. Keeping in mind the beneficial properties of these two nanoparticle groups, it was decided to incorporate 5 wt.% of TiO_2_ and ZrO_2_ in the EA and to probe the impact of their incorporation on different adhesive properties.

The SEM micrograph of TiO_2_ nanoparticles revealed that these particles were spherical in shape and present in an agglomerated form (Figure 1A). These findings are similar to a prior study that also demonstrated that TiO_2_ nanoparticles appear to be spherical in shape when observed under an SEM [35]. EDX mapping in the current study revealed the occurrence of Ti and oxygen (with Ti being more prevalent than oxygen, Figure 1B), which is again similar to the previous findings of Bhattacharya et al. [35]. The SEM micrograph of ZrO_2_ also led to comparable findings to previous studies [36,37], where Zr-based nanoparticles also demonstrated non-uniform morphology. EDX mapping for ZrO_2_ nanoparticles revealed the presence of Zr and oxygen, similar to previous studies [37,38].

In the present study, the rheological properties of all of the adhesives were also analyzed. The results demonstrated that, for all of the adhesives, a decrease in viscosity was observed when the angular frequency increased (Figure 3). This decrease in viscosity was more pronounced for the nanoparticle-containing adhesives as opposed to the EA. The results of this study are in line with an earlier study where an adhesive modified with nanofillers revealed lower viscosity, as compared with the control group [39]. Another former study also reported similar findings and associated decreased viscosity with the adhesive’s increasing filler content [40]. It should be kept in mind that the assessment of rheological properties is inconsistent and can be affected by the material’s handling [41], although the investigation of this characteristic was not part of this study.

In this study, half of the samples/groups selected for SBS testing were TC, while the remaining half remained NTC. The TC provided a vigorous challenge to the material in an in vitro setting which was identical to in vivo conditions. The ISO standard number 11,405 states that providing dental materials with a TC challenge with temperature ranges of 5–55 °C is appropriate to assess the aging of samples over a limited period of time [42]. We detected a reduced bond strength for all of the adhesives after they were TC, a result comparable to the formerly published literature [9,43]. The SBS testing experiments in this study revealed higher values for the nanoparticle-containing adhesives (with 5% TiO_2_ demonstrating the highest SBS values), as compared with the EA. These findings are similar to a former study that demonstrated an increase in the SBS of zirconia and TiO_2_ nanoparticle-containing adhesives [44]. TiO_2_ has been suggested as a reinforcing filler for dental materials due to its advantageous properties [45,46]. In addition, Zr-based nanoparticles disperse well in a material and improve its biocompatibility with organic polymers [47], which could have resulted in the improvement to the SBS observed in the present study. Another factor that could have played an important part in this finding could be the nano size of the two inorganic fillers used in this study. It is known that inorganic nanoparticles have high surface area [48] that can improve adhesion at the interface [49]. As the fillers were also nanosized, this could resulted in an improvement to the interfacial adhesion, resulting in greater SBS.

The bulk of the interfacial failures in this study were of the adhesive type (>70%). This finding is in line with the findings of an earlier study where adhesive-type failures were most commonly detected after the incorporation of filler nanoparticles in the composition of the adhesive [40]. It is pertinent to mention that dentin is collagenous in nature and this could have played a major part in the observation of adhesive-type failures in the current study, as the bonding of adhesives to dentin is challenging and is still considered a puzzling task in dentistry [50].

The resin–dentin interface was also analyzed (via SEM) in this study. It was witnessed that hybrid layers and resin tags (of variable depth) were formed for the EA (Figure 4A) and the 5% TiO_2_ group (Figure 4B), whereas the 5% ZrO_2_ group demonstrated compromised hybrid layer and resin tag formation (Figure 4C). Earlier studies demonstrated that the thickness of the adhesive layer and the length of the resin tag are not essential determinants of the bond strength, which is actually dependent on the adhesive’s composition [51,52]. Therefore, we still advocate the addition of ZrO_2_ nanoparticles to adhesives due to the improved SBS and comparable rheological properties to the other adhesive groups observed in the current study.

Although the results of the present study are encouraging, readers should exercise caution while interpreting them. The outcomes are possibly limited to the particular adhesive fabricated in the study and thus may not be applicable to commercially available adhesives. In addition, the TiO_2_ and ZrO_2_ contents were arbitrarily determined; however, different content percentages of these nanoparticles may show different properties. Moreover, the SBS test is a static test for bond strength while, clinically, material interfaces are commonly exposed to fatigue loads, which could influence the bond strength and durability of modified adhesives. We observed an improved SBS for the two nanoparticle-containing adhesives; however, both demonstrated lower viscosity compared with the EA. Future studies probing the impact of the integration of various other concentrations of these nanoparticle groups are recommended. We suggest further exploration of the effect of the incorporation of 2.5 wt.% TiO_2_ and ZrO_2_ on the mechanical properties of adhesives to further improve understanding of the relationship between filler concentrations and SBS and rheological properties.

## 5. Conclusions

Reinforcing the EA with filler nanoparticles (TiO_2_ and ZrO_2_) caused an increase in the adhesive’s SBS and the 5% TiO_2_ group demonstrated the highest values. The SBS values for the 5% TiO_2_ group were significantly different from the EA but were non-significantly different from the 5% ZrO_2_ group (for both NTC and TC samples). In addition, both nanoparticle-containing adhesive groups demonstrated comparable viscosity to the EA; nevertheless, their values were less than that of the control group. Additional studies examining the impact of diverse filler concentrations on the various properties of adhesives are suggested.

## Figures and Tables

**Table 1:** Shear-bond strength (SBS) and failure mode analysis among the study groups.

	SBS (MPa) (Mean ± SD)			Failure Mode Analysis (%)		
Group (*n* = 10)	NTC	TC	*p*-Value *	Adhesive	Cohesive	Mixed
Control EA (0% particles)	21.03 ± 2.44 ^a,A^	-	<0.01	100	0	0
	-	17.62 ± 1.70 ^a,B^		100	0	0
5% TiO_2_	25.35 ± 1.53 ^b,A^			80	0	20
	-	23.89 ± 1.95 ^b,B^		100	0	0
5% ZrO_2_	23.10 ± 2.22 ^b,A^			70	10	20
	-	20.72 ± 1.32 ^b,B^		80	0	20

TC: thermocycling, NTC: no thermocycling, TiO_2_: titanium oxide, ZrO_2_: zirconium oxide, Control EA, experimental adhesive with no nanofiller composite. * ANOVA. Dissimilar lower case letters in the same column indicate statistical significance. Dissimilar capital letters in a row (same group) indicate statistical significance.

## Data Availability

The data analyzed in this study are available on request form the corresponding author.
